# Effect of vanadium (V) doping on the physical characteristics of novel n-type TiO_2_ for sustainable energy storage

**DOI:** 10.1039/d5ra08419a

**Published:** 2025-12-03

**Authors:** A. Timoumi, Ziad Moussa, Hatem M. Altass, Khalid Althagafy, Abdulrahman A. Alsimaree, Munirah M. Al-Rooqi, Rabab S. Jassas, Saleh A. Ahmed

**Affiliations:** a Department of Physics, Faculty of Science, Umm Al-Qura University 24382 Makkah Saudi Arabia aotemoume@uqu.edu.sa; b Department of Chemistry, College of Science, United Arab Emirates University P. O. Box 15551 Al Ain United Arab Emirates; c Department of Chemistry, Faculty of Science, Umm Al-Qura University 21955 Makkah Saudi Arabia; d Department of Chemistry, College of Science and Humanities, Shaqra University Shaqra Saudi Arabia; e Department of Chemistry, Jamoum University College, Umm Al-Qura University 21955 Makkah Saudi Arabia; f Chemistry Department, Faculty of Science, Assiut University 71516 Assiut Egypt saahmed@uqu.edu.sa saleh_63@hotmail.com

## Abstract

The design of high-performance advanced materials for green energy nanotechnology is vital for progress in cutting-edge domains such as environmental sustainability and energy conversion technologies. In this study, we report the synthesis and characterization of undoped and vanadium-doped titanium dioxide (TiO_2_) in both pellet and thin film forms. The influence of vanadium doping levels on the material's physical and electrical properties was systematically investigated. Structural, morphological, compositional, electrical, and optical analyses were performed using X-ray diffraction (XRD), scanning electron microscopy (SEM), X-ray photoelectron spectroscopy (XPS), impedance spectroscopy (IS) (for pellets) and UV-visible spectroscopy (for films). XRD results confirmed that all samples were polycrystalline in the anatase phase, while increasing vanadium content reduced crystallite size and enhanced density. While the elemental composition remained relatively stable, XPS data showed the formation of Ti^3+^ states and oxygen vacancies, which play a pivotal role in modifying electronic behavior. Impedance spectroscopy indicated semiconducting behavior, with resistance decreasing as temperature increased, reflecting enhanced conductivity. Optical studies (UV-vis spectroscopy) showed a redshift in the absorption edge toward the visible region, with a reduction in bandgap energy from 3.20 eV (undoped) to 2.85 eV (6% V-doped), attributed to localized states and the formation of impurity bands. Electrical measurements showed enhanced conductivity with temperature and a clear transition to non-Debye behavior, reflecting a distribution of relaxation times likely due to grain boundaries and defect states. Notably, the dielectric constant was significantly elevated, supporting potential use in energy storage or solar energy applications. This work provides key insights into tailoring TiO_2_-based materials through metal doping for sustainable energy storage.

## Abstract 2

The design of high-performance advanced materials for green energy nanotechnology is vital for progress in cutting-edge domains such as environmental sustainability and energy conversion technologies.

## Graphical abstract

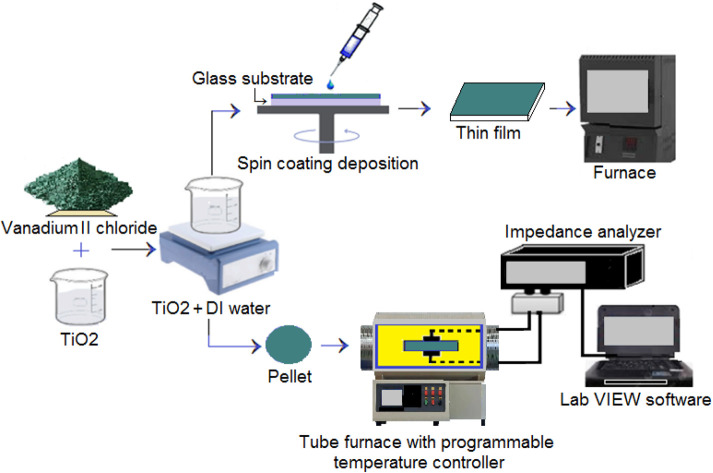

## 1. Introduction

The evolution of solar cell technologies is broadly categorized into three main generations: silicon-based, thin-film, and organic solar cells.^1–3^ Among these, thin-film solar cells have reached commercial maturity, with global production reaching 30 GW. Despite this progress, ongoing research is driven by the need to develop solar cells that are not only highly efficient but also cost-effective and environmentally sustainable.^4^ Transparent conducting oxide (TCO) materials have drawn considerable attention due to their favorable physical, chemical, optical, and optoelectronic properties. Among these, titanium dioxide (TiO_2_) is chosen, owing to its high refractive index, chemical stability, low cost, non-toxicity, and excellent photocatalytic activity.^5^ Thin films of TiO_2_ are especially advantageous in electronic, photonic, and optoelectronic applications, as the material properties at reduced dimensions often outperform their bulk counterparts.^6^ TiO_2_ exists in several polymorphic forms, primarily anatase, rutile, and brookite, with direct optical bandgaps of approximately 3.2 eV, 3.4 eV, and 3.37 eV, respectively.^7^ Among these, the anatase phase is generally considered the most photoactive for photocatalytic applications.^8^ The deposition of TiO_2_ thin films is fundamental across various technological fields, and numerous fabrication methods have been developed to tailor these films for specific applications.^9–13^ Doping TiO_2_ with metal ions is a well-established strategy to tune its interfacial electron transport and enhance photoreactivity.^12–14^ Metal ion doping, such as with Fe, Ag, Zn, Cu, or V has been shown to significantly modify the structural, optical, and electrical properties of TiO_2_ thin films.^15–20^ In particular, dopants like Nb, Fe, and Zn are known to reduce TiO_2_'s wide bandgap, making it responsive to visible light, a key requirement for solar energy conversion.^21–23^ Doping TiO_2_ with transition elements having high oxidation states (such us; Mo^6+^, Nb^5+^, W^6+^, V^5+^) shows good photo catalytic characteristics than doping with transition elements having lower oxidation states (such us; Fe^3+^, Co^2+^, Ni^2+^). This is the result of a larger number of hydroxyl groups on the surface, which makes such surfaces more photocatalytically active.

Among these dopants, vanadium (V) remains relatively underexplored. Due to its similar ionic radius to titanium, V is a suitable dopant that can substitute into Ti sites in the TiO_2_ lattice.^24^ Vanadium enhances visible light absorption^25^ and facilitates charge separation by reducing charge–hole recombination. Furthermore, it may assist in the transport of photo-excited electrons across the surface, thus enhancing the material's photocatalytic and optoelectronic performance.

Vanadium may be readily doped into TiO_2_ because its ionic radius is nearly identical to that of titanium.^26^ As a result, numerous scientists have created V-doped TiO_2_ and investigated its photocatalytic characteristics. According to Klosek and Raftery,^26^ ethanol can be photodegraded by V-doped TiO_2_ when exposed to visible light. TiO_2_ catalysts were altered by Anpo *et al.*^27^ by subjecting them to high energy metal ion bombardment. The V ion was more effective in the red shift, and the metal ion-implanted TiO_2_ exhibited a significant absorption shift toward the visible light band. V-doped TiO_2_ with visible activity was created by Zhou *et al.*^28^ using ion implantation and modified sol–gel techniques, respectively. Although vanadium and vanadium-based compounds have several advantages in energy storage and photocatalysis, there are several drawbacks, mainly with regard to mass transport constraints, structural stability, and charge carrier recombination.^29^

Herein, we intend to clarify the role of vanadium in the structural, the morphological, the optical, and the electronic properties of undoped and vanadium-doped TiO_2_, with particular focus on the influence of varying vanadium amounts. Commercially available TiO_2_ powder was doped with vanadium and separately processed into pellets and thin films for further investigation. Through obtained detailed characterization, we assessed the feasibility of using these readily available samples as a practical and economical source material. To the best of our knowledge, this work introduces also a novel and efficient route for optimizing thin film fabrication conditions for physical vapor deposition (PVD)-based applications or sustainable energy storage.

## 2. Materials, synthesis and characterizations

### 2.1 Materials

Titanium dioxide (TiO_2_, 99.5% purity, CAS No. 13463-67-7) and vanadium(ii) chloride (VCl_2_, 99% purity, CAS No. 10580-52-6) were purchased from Sigma-Aldrich. For pellet formation, the powders were thoroughly mixed and compressed into cylindrical pellets (8 mm diameter) using a stainless-steel mold equipped with a punch and counter-punch to ensure uniform compaction.

### 2.2 Synthesis of thin films and pellets samples

TiO_2_ powder (5 g) was dispersed in 50 mL of deionized water and stirred for 30 minutes at room temperature (27 °C) to form a uniformly dispersed white suspension. Vanadium(ii) chloride was then added in varying amounts (0.0, 0.2, 0.3, and 0.4 g) to obtain doped samples. The V-doping content in TiO_2_ is expressed as a percentage of weight of the base amount of titanium oxide, which is 5 grams. The following ratios represent the proportion of VCl_2_ to the basic amount of TiO_2_ (0%, 4%, 6% and 8%). The activator content (V) in TiO_2_ is expressed as a percentage by weight of the base amount of titanium oxide, which is 5 grams.

**Fig. 1:**
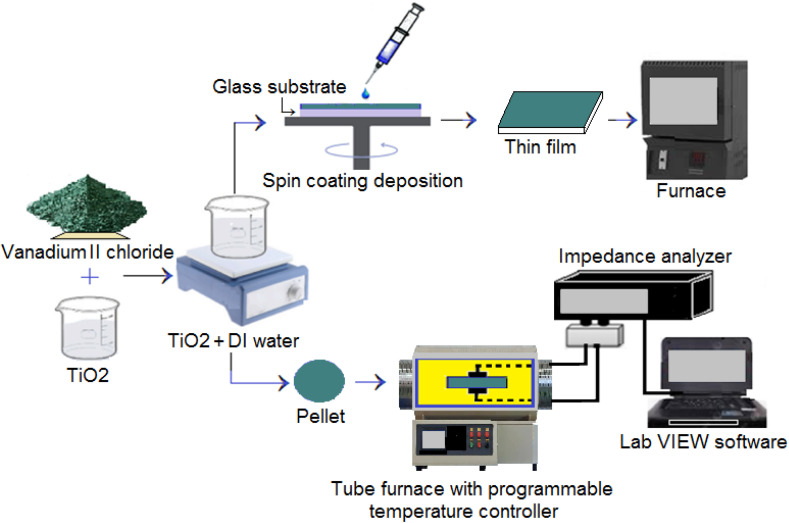
Schematic diagram illustrating the setup of the impedance spectroscopy device.

The resulting mixtures were sonicated to enhance homogeneity and then applied to clean glass substrates using a spin coating process. Each layer was deposited by spinning at 6000 rpm for 30 seconds, followed by a brief drying period using mild heating. Multiple layers were applied in a stepwise manner to achieve uniform film coverage. After that, samples are annealed on hot plate at 300 °C for about 15 min. The average final thickness is estimated to be about 300 nm for all samples. The acquired samples are nearly homogeneous and uniform. Therefore, the deposition technique and doping process which can be impacted by elements like dopant concentration and precursor solution stability determine the uniformity of doped films. For pellet, the dried mixtures were pressed into mold (stainless steel with punch and counter-punch) using a uniaxial hydraulic press at 6000 psi (41.4 MPa) for a few minutes. Each sample was compressed into a pellet with a diameter of 12 mm and a thickness of 1 mm, measured using a Vernier caliper. A total of four distinct sample sets were prepared. Pellets are placed in a programmable furnace allowing the temperature to be adjusted and fixed during measurements of the electrical characteristics. The atmosphere of the furnace is air and the heating time of the pellets to measure the results is almost 30 minutes. The overall synthesis process is illustrated in Fig. 1.

### 2.3 Methods of characterization

The crystal structure of the samples was analyzed using X-ray diffraction (XRD) with a Unisantis XRD-300 analyzer, employing Cu-Kα radiation (*λ* = 0.15406 nm) and a graphite monochromator. Scans were performed over a 2*θ* range of 10° to 70°. X-ray photoelectron spectroscopy (XPS) was conducted using a JPS-9030 spectrometer equipped with an Al-Kα X-ray source (photon energy = 1487 eV). All binding energies were referenced to the C 1s peak at 284.6 eV. Surface morphology was examined using scanning electron microscopy (SEM). The quantitative elemental composition of the films was analyzed *via* EDX. Optical properties were evaluated at room temperature using a UVD-2950 double-beam UV-Vis spectrophotometer over the wavelength range of 200–1100 nm. Electrical AC conductance was measured using the setup shown in Fig. 1. Frequency-dependent conductivity measurements were performed at ambient temperature, while temperature-dependent measurements were conducted in the range of 280 °C to 500 °C, in 20 °C increments. An Agilent 4294A impedance analyzer was used to record conductance after thermal equilibrium was reached at each step, with a temperature tolerance of ±2 °C. LabVIEW software was used to control experimental parameters and collect data, except for the temperature control which was *via* the programmable oven.

## 3 Results and discussion

### 3.1 Diffraction measurements of X-rays

**Fig. 2:**
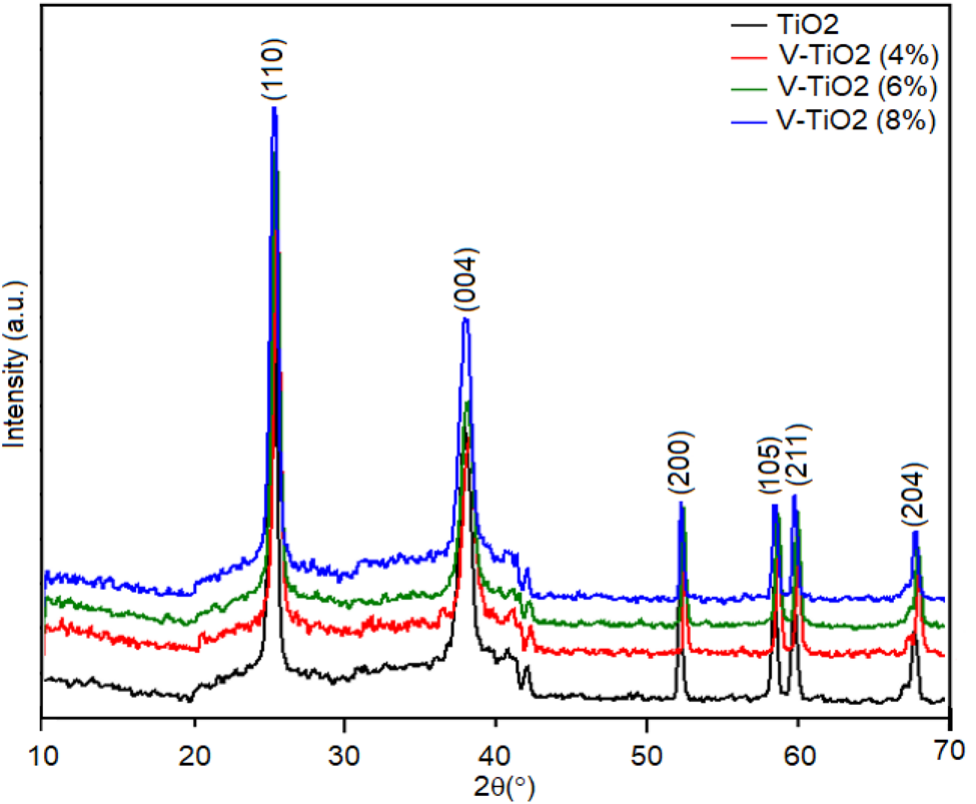
X-ray diffraction (XRD) patterns of undoped and V-doped TiO_2_ samples with varying vanadium-doping levels.

The crystallographic properties of undoped and vanadium-doped TiO_2_ samples were analyzed using X-ray diffraction (XRD) over a 2*θ* scan range of 10° to 70°, as shown in Fig. 2. The diffraction patterns of all samples closely matched the standard pattern of the anatase phase of TiO_2_, indicating that the fundamental crystal structure remained unaltered by doping. No additional peaks corresponding to secondary phases or vanadium oxides were detected, suggesting successful incorporation of vanadium without significant structural disruption. Using the Scherrer equation, the average crystallite size (*D*) of the films was estimated from the XRD data:^30^1
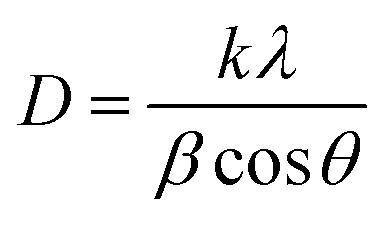
where *D* is the crystallite size, *k* is the shape factor, *λ* is the X-ray wavelength, *β* is the full width at half maximum (FWHM) of the diffraction peak, and *θ* is the Bragg angle. The estimation was based on the FWHM of the (110) diffraction peak and yielded an average crystallite size of approximately 14 nm. The slight reduction in crystallite size upon doping can be attributed to the presence of vanadium, which inhibits grain growth during the nitrogen adsorption measurements^31^ and the surface becomes more densely packed. This tendency, which is associated with the addition of V^3+^ ions to the TiO_2_ lattice, causes lattice strain and defects, which result in the creation of finer grains.

Furthermore, the XRD patterns revealed minor shifts in 2*θ* values with increasing dopant concentrations, suggesting lattice distortion caused by the incorporation of vanadium ions into the TiO_2_ crystal lattice. This substitution may influence the crystal structure and induce strain. Enhanced structural integrity and increased carrier mobility observed in doped TiO_2_ samples indicate that vanadium doping not only affects crystallite size but also contributes to improved thermal and electronic stability.^32^ Reducing crystallite size improves film thickness, boosts photocatalytic activity, and prevents photo-excited electron–hole pair recombination. These findings suggest that vanadium incorporation enhances both structural and functional properties of TiO_2_.

According to JCPDS Card No. 65-5714, the XRD peaks observed at 25.50°, 37.81°, 51.91°, 58.61°, 59.71°, and 67.32° represent the (110), (004), (200), (105), (211), and (204) planes of the anatase phase of TiO_2_, respectively. These characteristic reflections confirm that both the undoped and V-doped TiO_2_ samples maintain the anatase crystal structure. Vanadium segregation at grain boundaries could be the cause of the lack of subsequent phases. This implies that vanadium considerably disturbs the normal crystal structure at higher activation levels, resulting in a more intricate and uneven surface shape.

### 3.2 Surface morphology testing

#### 3.2.1 SEM analysis

**Fig. 3:**
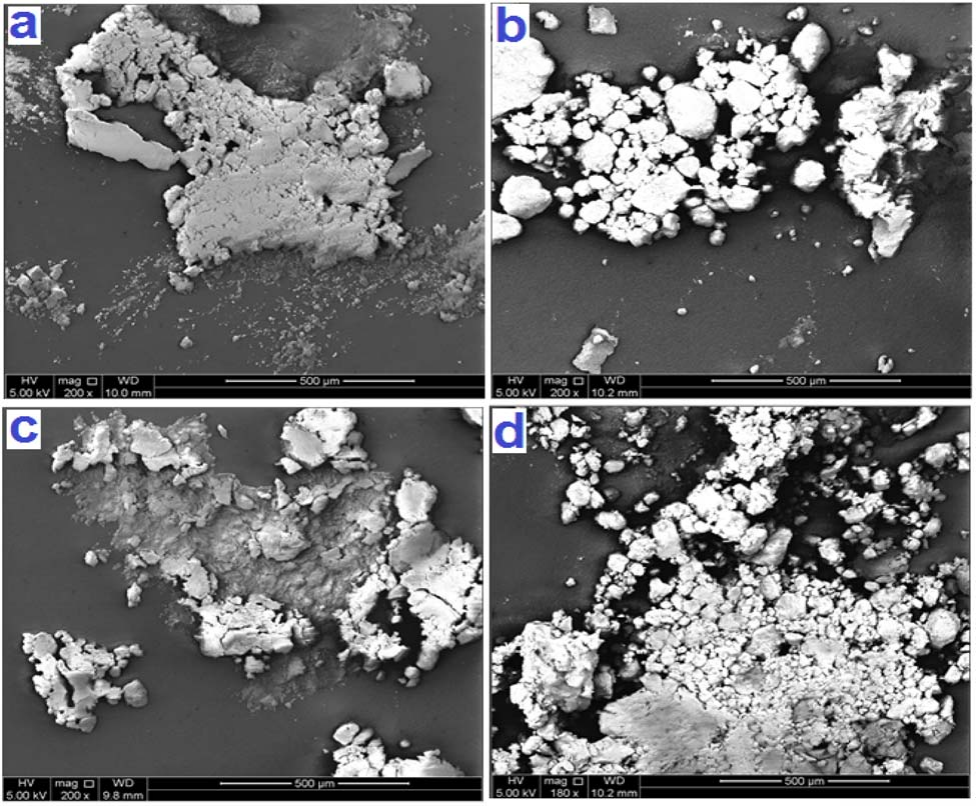
SEM images of (a) undoped TiO_2_, (b) 4V-doped TiO_2_, (c) 6V-doped TiO_2_, and (d) 8V-doped TiO_2_ samples.

SEM images were collected to investigate the morphology of the synthesized samples. As shown in Fig. 3, the samples primarily consist of aggregated nanoparticles. The surface roughness has significantly increased in this figure. The incorporation of vanadium alter the surface morphology. The SEM images of undoped TiO_2_ (Fig. 3a) and V-doped samples 4%, 6%, and 8% (Fig. 3b–d, respectively), exhibit comparable nanoparticle morphology, indicating that vanadium doping impact the overall structural appearance of the material. Thus, at high doping levels, particle aggregation could occur. High doping levels can cause phase segregation, decreased solubility, and the development of dopant-rich domains or bigger aggregates in a variety of materials, especially organic semiconductors and solutions. The space charge layer width can be decreased by high doping, but this effect can be reversed by nanostructuring, enabling volume fractions that are comparable to those of larger, low-doped particles. In applications like photocatalysis and batteries, this can enhance overall performance.

#### 3.2.2 EDX analysis

The materials' quantitative composition was examined using energy dispersive X-ray analysis (EDX). The atomic percentages for each sample are displayed in Table 1, which indicates that V atoms are present in the TiO_2_ lattice.

**Table 1:** Atomic composition of samples

Sample	Mass atomic (%)		
	Ti	O	V
TiO_2_	13.93	86.07	—
4% V-doped TiO_2_	10.46	88.78	0.63
6% V-doped TiO_2_	12.37	85.38	1.79
8% V-doped TiO_2_	11.98	83.92	3.49

### 3.3 XPS analysis

**Fig. 4:**
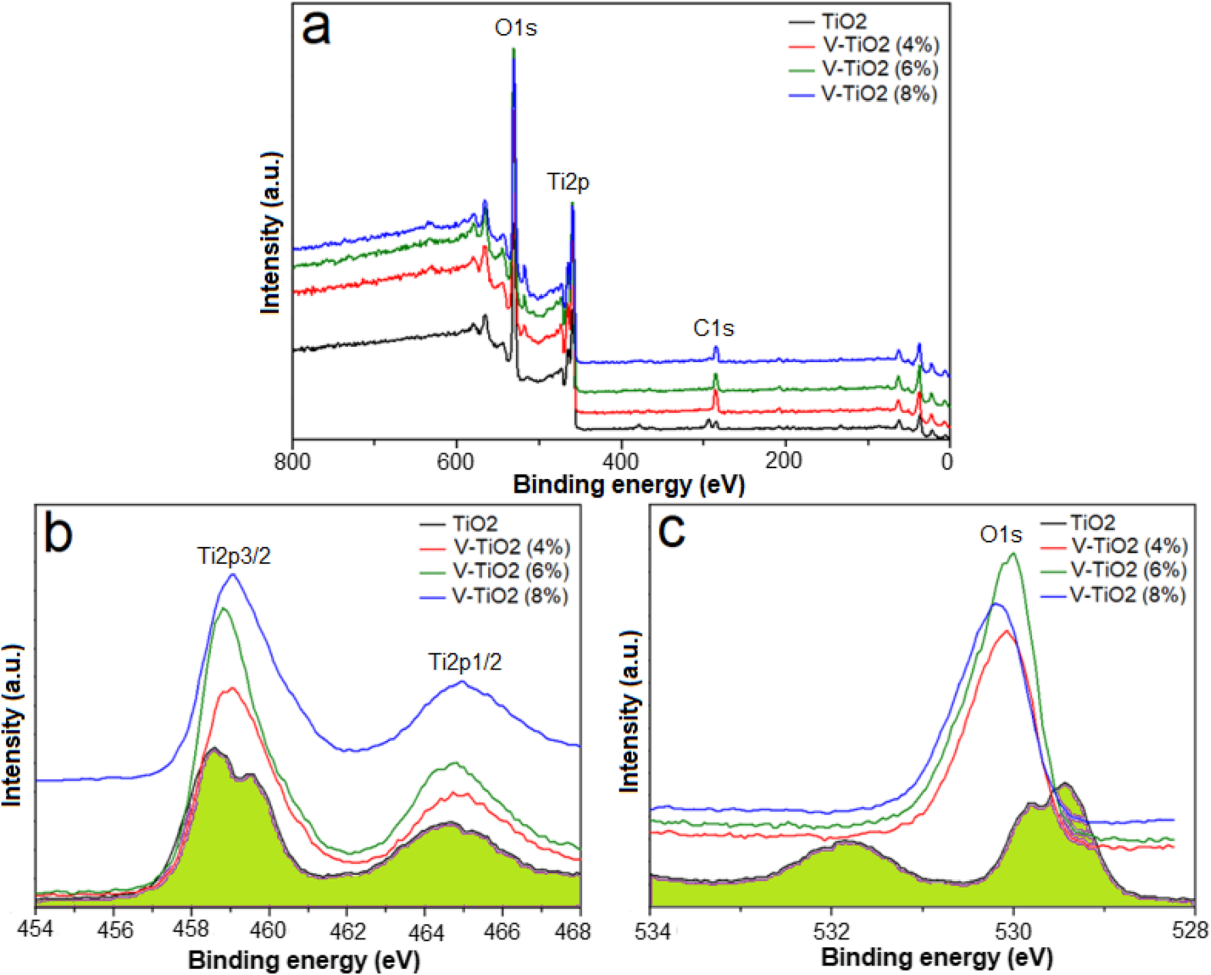
(a) XPS survey spectra of pure TiO_2_ and V-doped TiO_2_ samples, showing the elemental composition, and high-resolution spectra of (b) Ti 2p peaks and (c) O 1s peaks.

The elemental compositions and chemical states of each constituent element were investigated using X-ray Photoelectron Spectroscopy (XPS). The XPS measurements revealed the surface chemical composition and valence states. Fig. 4 displays the full survey spectrum of the pure TiO_2_ sample. Several peaks appear within the 0–800 eV binding energy range. The XPS survey confirmed the presence of titanium (Ti), oxygen (O), and carbon (C) in the sample. The C 1s peak, consistently observed at 284.6 eV, is usually attributed to the adventitious carbon or carbon residues from the organic precursor. All samples showed distinct peaks corresponding to C 1s, O 1s, and Ti 2p. The high-resolution Ti 2p spectrum of the undoped TiO_2_ (Fig. 4a) exhibited characteristic Ti^4+^ signals at ∼458 eV (Ti 2p_3/2_) and ∼464 eV (Ti 2p_1/2_), confirming the anatase phase. In doped samples, a slight shift in the Ti 2p peaks toward higher binding energies was observed, which may be attributed to partial reduction of Ti^4+^ due to localized electrons at oxygen vacancy (V_o_) sites.^33,34^ Additionally, peak broadening in the Ti 2p region for doped samples indicates the presence of smaller crystallites, consistent with XRD results.

Fig. 4b displays the high-resolution spectrum of Ti 2p. The spin–orbit splitting of this doublet results in two distinct peaks: Ti 2p_3/2_ (binding energy at 459.8 eV) and Ti 2p_1/2_ (binding energy at 465.5 eV), corresponding to the Ti^4+^ oxidation state in the TiO_2_ lattice.^35^Fig. 4c shows the high-resolution O 1s spectra for the samples, exhibiting two main components. The lattice oxygen (O^2−^) in TiO_2_ is represented by the peak at ∼530 eV, while surface hydroxyl groups (Ti–OH) or adsorbed oxygen species are responsible for the peak at 538 eV.^36,37^ These surface-related oxygen species are crucial for photocatalysis, as they can trap photogenerated holes and facilitate the formation of highly reactive hydroxyl radicals, which play a significant role in the degradation of organic contaminants.^38^ The presence of peak in the TiO_2_ sample at 529.5 eV suggests the existence of oxygen vacancies (V_o_) and surface Ti–OH species, which enhance photocatalytic activity by improving charge separation and extending visible-light absorption.^39,40^ These oxygen-related defects are therefore crucial contributors to the observed photocatalytic behavior of the pure and doped TiO_2_ powder. By creating defect states that reduce the band gap, allowing visible light absorption and offering locations for charge carrier separation, the creation of Ti^3+^ and oxygen vacancies (OVs) in TiO_2_ enhances photocatalytic and electrical activity.

### 3.4 Analysis of electrical characteristics

**Fig. 5:**
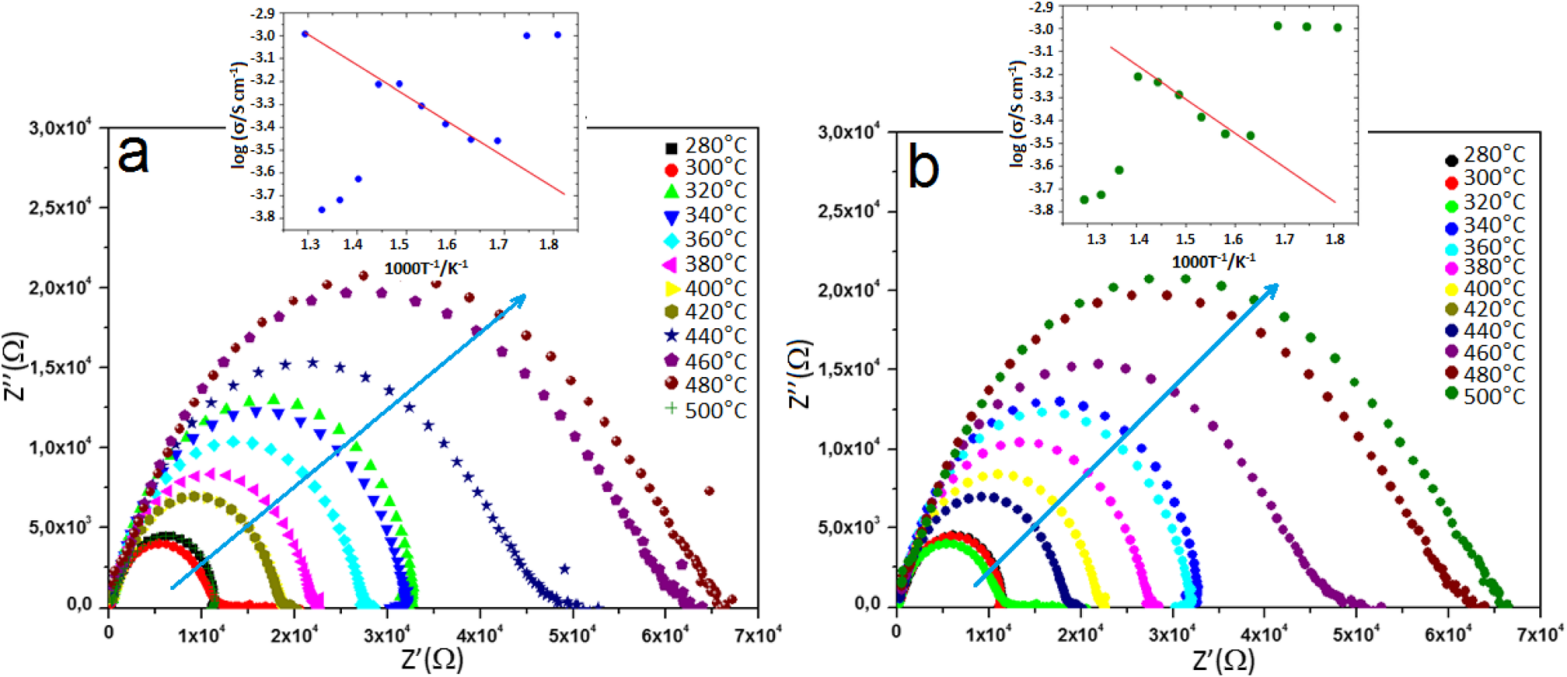
Nyquist plots (*Z*″ *vs. Z*′) showing the impedance response of (a) undoped and (b) 8% V-doped TiO_2_ pellets.

Impedance spectroscopy (IS) was employed to investigate the dielectric behavior of pure and V-doped TiO_2_ samples as a function of temperature. To enable measurements, the pellets were coated with electrodes connected to copper wires. The Nyquist diagrams (*Z*″ *vs. Z*′) reveal a decline in resistance with increasing temperature, which can be attributed to the thermal activation of charge carriers. A decrease in the semicircular arc width at elevated temperatures indicates enhanced conductivity.^41^ Moreover, variations in the width and shape of the semicircles with temperature are characteristic of typical semiconducting behavior.^42^ To determine whether the observed abnormal dielectric relaxation (ADR)^43^ is associated with the positive temperature coefficient of resistance (PTCR) effect due to a metal–insulator transition (MIT) influenced by humidity,^44^ Nyquist plots were recorded, as shown in Fig. 5. In these plots, *Z*′ and *Z*″ represent the real and imaginary components of the complex impedance (Z*), respectively. The Nyquist diagrams exhibit distinct features for undoped and V-doped TiO_2_. The large semicircular arc in the high-frequency region, which ends at the origin, corresponds to the bulk (grain) response. In contrast, a short linear tail in the low-frequency region represents the interfacial or electrode polarization response. This tail is attributed to Warburg impedance.^45^ The Warburg component becomes more pronounced at 300 °C and diminishes at higher temperatures, consistent with the desorption of water molecules from the surface. As temperature increases, the main semicircular arc expands, indicating PTCR behavior.^46^ The impedance response can be modeled by an equivalent circuit consisting of a resistor (*R*) and a capacitor (*C*) connected in parallel.^47^ For such a system, the imaginary component of the impedance is given by:2
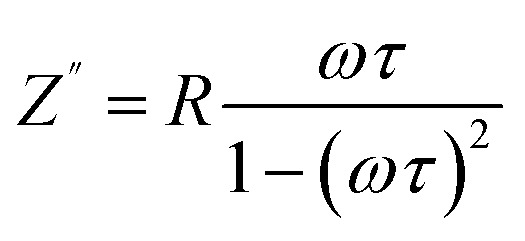
where the relaxing time is indicated by *τ* = *RC* and the angular frequency by *ω* = 2π*f*. At the frequency where *ωτ* = 1, a peak with an intensity of *R*/2 is observed in the imaginary section of the impedance. The Nyquist plots can also be used to extract the resistance and relaxation time values at different temperatures, providing insights into the electrical conduction mechanisms and relaxation phenomena of the materials.

The experimental values of electrochemical conductivity (*σ*) were calculated for both the TiO_2_ and V-TiO_2_ samples (pellet form) using the eqn (3). In this equation, *L* represents the pellet thickness, *S* denotes the area of the silver electrode, and *R* stands for the fitted resistance value from Nyquist plots.

3 *σ* = *L*/*SR*

**Fig. 6:**
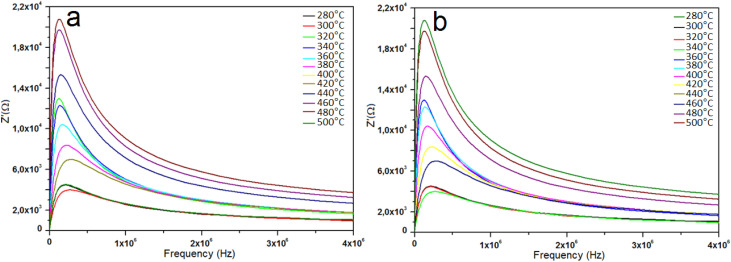
Real part of impedance (*Z*′) *vs.* frequency of (a) undoped and (b) 8% V-doped TiO_2_ pellets.

The variation of the real part of impedance (*Z*′) with frequency at different temperatures is shown in Fig. 6. At lower frequencies, *Z*′ increases due to dominant polarization effects, which lead to higher resistance.^48,49^ Because ions and electrons move more readily at higher temperatures, resistance typically decreases. Temperature influences impedance by affecting the speed of chemical and physical processes. Impedance, particularly the real component, varies throughout the spectrum, with low- and mid-frequency regions frequently displaying a larger reliance on temperature. As the temperature rises, thermal activation of charge carriers results in a decrease in *Z*′, particularly at higher frequencies where the values tend to converge. This behavior indicates enhanced conductivity with increasing temperature and is characteristic of semiconducting materials. Impedance analysis revealed thermally stimulated conduction and non-Debye behavior (non-exponential relaxation processes). Defect states and particle/grain boundaries can be linked to this occurrence. Particle and grain boundaries may cause non-ideal relaxation because of differences in conductivity and resistance between the bulk material and the boundaries.

**Fig. 7:**
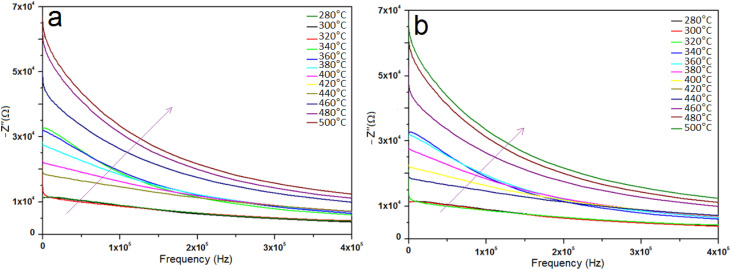
Imaginary part of impedance (*Z*″) *vs.* frequency of (a) undoped and (b) 8% V-doped TiO_2_ pellets.

The variation of the imaginary part of impedance (*Z*″) with frequency at different temperatures is shown in Fig. 7. As frequency increases, *Z*″ initially rises, reaching a maximum at a characteristic relaxation frequency, then decreases. This peak corresponds to the relaxation time of the system. With increasing temperature, the *Z*″ values decrease, indicating a reduction in resistance due to enhanced charge carrier mobility. The observed shift in the relaxation peak with temperature reflects the influence of thermal activation on the electrical relaxation behavior of the samples.^50–52^

### 3.5 Optical analysis of V-TiO_2_ thin films

**Fig. 8:**
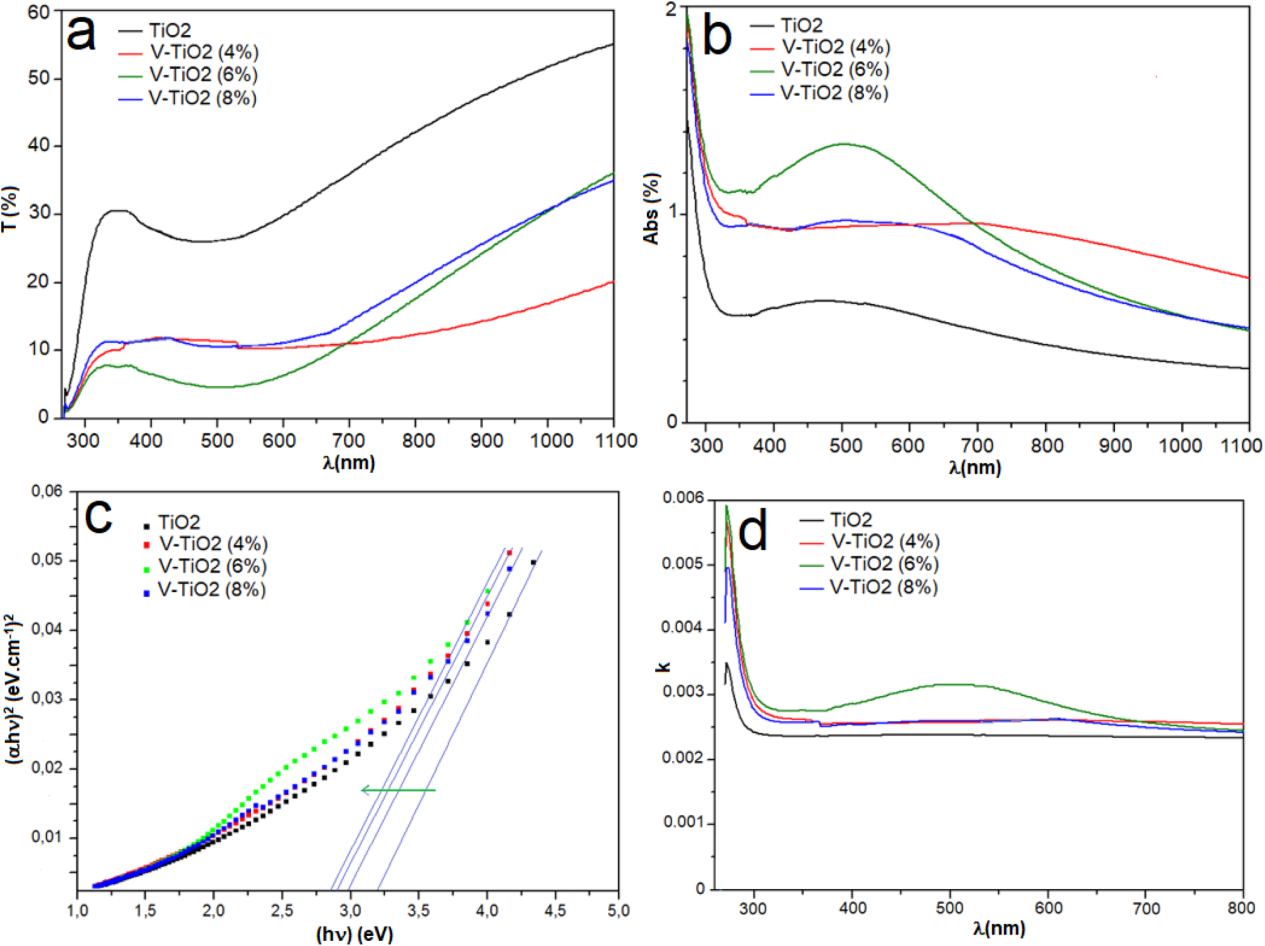
Optical properties of undoped and vanadium-doped TiO_2_ thin films: (a) transmission spectra, (b) absorbance spectra, (c) Tauc plots for bandgap estimation, and (d) variation of the extinction coefficient (*k*).

Understanding the optical characteristics of materials is necessary for the design and assessment of optoelectronic devices. To assess the impact of vanadium incorporation on the optical behavior of TiO_2_ thin films, we investigated their transmittance and absorbance spectra across the wavelength range of 250–1100 nm. Fig. 8a illustrates the optical transmittance profiles of both undoped and V-doped TiO_2_ films at various doping levels. The undoped films exhibited strong absorption in the UV and visible regions (250–600 nm), with negligible transmittance, indicating minimal photon penetration. Upon V doping, the absorption edge initially shifted toward shorter wavelengths (blue shift), which is attributed to the Burstein–Moss effect.^53^ Under these conditions, the introduction of vanadium increases the density of free carriers. Vanadium atoms introduce localized states near the conduction band, increasing electronic band tailing and promoting sub-bandgap absorption, thereby narrowing the effective optical bandgap. Additionally, V doping had a pronounced effect on transmittance. An initial improvement in transparency was observed, particularly in the visible region, where transmittance exceeded 50% at an optimal doping level. At higher doping concentrations, however, transmittance declined significantly (down to ∼15%), likely due to increased light scattering and absorption from defects, grain boundaries, or carrier-induced intraband transitions. This suggests that structural or electrical alterations brought on by excessive doping may impair optical transparency. Film thickness affects the ratio of optical transparency to light absorption, which has an impact on solar cells and energy storage devices. Fig. 8b shows the absorbance spectra of the films. The observed increase in absorbance with doping may stem from changes in the electronic structure or the formation of defect states induced by V incorporation.

The energy bandgap of the TiO_2_ thin films was estimated using the Tauc method, as illustrated in Fig. 8c. The Tauc relation is expressed by the following equation:^54,55^

4 (*αhν*) = *A*(*hν* − *E*_g_)^*n*^

where *n* = 1/2 for direct allowed transitions and *n* = 2 for indirect transitions, *A* is a material-specific constant, *h* is Planck's constant, and *α* is the absorption coefficient, calculated using:^56,57^

5 *α* = (1/*e*){ln(1 − *R*)^2^/*T*}

The values of the resulting optical bandgap are given in Table 2.

**Table 2:** Optical bandgaps of undoped and V-doped TiO_2_ thin films at various doping levels

Thin layers	Gap energy (eV)
TiO_2_	3.20
4% V-doped TiO_2_	2.90
6% V-doped TiO_2_	2.85
8% V-doped TiO_2_	3.00

A reduction in the bandgap energy is observed with increasing vanadium content. This narrowing is likely caused by the formation of oxygen vacancies and localized states associated with Ti^3+^ ions.^58–60^ When oxygen vacancies occur, electrons localize around the vacancy sites, creating donor levels just below the conduction band. These Ti^3+^-related states introduce intermediate energy levels within the bandgap. Transition metal doping, such as with vanadium, can further contribute to bandgap narrowing through lattice distortion and defect formation, including oxygen vacancies and impurity states.^61^ This effect is clearly demonstrated by the reduced bandgap values with increasing dopant levels. A photocatalyst's activity under solar irradiation is greatly increased when its bandgap is decreased, which makes it possible for it to absorb visible light more effectively. However, other material parameters such as particle size, morphology, and surface area also play a role in modulating the bandgap in semiconductor materials.

Fig. 8d shows the variation of the extinction coefficient (*k*) with wavelength in the 250–800 nm range for both undoped and V-doped TiO_2_ films. The extinction coefficient was determined using the relation:^62^

6 *K* = (*αλ*/4π)

**Fig. 9:**
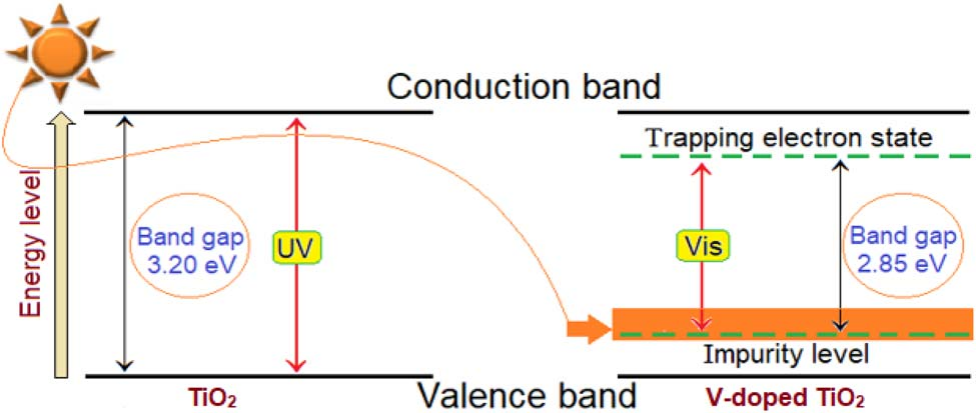
Hypothetical optical band gap of undoped and doped V-TiO_2_ thin films.

As evident from the figure, the extinction coefficient increases in the 330–800 nm range for both pure and doped samples. The introduction of vanadium dopants is further enhanced, reflecting a corresponding rise in the absorption coefficient. This increase is attributed to the creation of shallow trap states within the band structure due to vanadium incorporation, which promotes additional photon absorption and thus lowers the optical bandgap, as illustrated in Fig. 9.^63^

Developing composite materials or engineering defects to improve performance and stability are two ways to overcome the limitations of vanadium doping and its application under circumstances. High-level theoretical computations can be investigated, novel materials can be designed using machine learning, and new structures such as ultrathin or two-dimensional vanadium oxides can be created.

## 4 Conclusion

This study investigated the effects of vanadium doping on the structural, optical, and dielectric properties of TiO_2_ thin films. XRD confirmed the anatase phase with enhanced crystallinity and reduced crystallite size upon doping. XPS analysis indicated the formation of Ti^3+^ states and oxygen vacancies, which play a key role in modifying optical and electronic behavior. SEM images showed uniform, spherical nanoparticles with increasing homogeneity at higher doping levels. Optical studies revealed a bandgap narrowing from 3.20 eV (undoped) to 2.85 eV (6% V-doped), attributed to intermediate states and increased disorder. The extinction coefficient increased across the visible range, reflecting enhanced light absorption. Dielectric measurements showed a high dielectric constant at low frequencies, influenced by porosity and space charge effects. Impedance analysis indicated thermally activated conduction and non-Debye behavior. Overall, vanadium doping effectively tunes the optoelectronic properties of TiO_2_, making it a strong candidate for energy storage and photocatalytic applications. To further improve the performance of TiO_2_ materials, further study could focus on co-doping, preparation process optimization, or classical simulations.

## Author contributions

All authors contributed to conceptualization, methodology, writing original draft, writing review & editing, and formal techniques to analyze or synthesize study data.

## Conflicts of interest

There are no conflicts to declare.

## Data Availability

This is to certify that all required and available data supported the work are included in the manuscript as figures and tables.
